# Automation at the service of the study of executive functions in preclinical models

**DOI:** 10.1038/s41598-023-43631-8

**Published:** 2023-10-06

**Authors:** Francesca Zoratto, Edoardo Pisa, Claudia Soldati, Caterina Barezzi, Angela Maria Ottomana, Martina Presta, Valerio Santangelo, Simone Macrì

**Affiliations:** 1 Centre for Behavioural Sciences and Mental Health, Istituto Superiore di Sanità, Rome, Italy; 2 Neuroscience Unit, Department of Medicine, University of Parma, Parma, Italy; 3 Department of Physiology and Pharmacology “Vittorio Erspamer”, “Sapienza” University of Rome, Rome, Italy; 4 Department of Philosophy, Social Sciences and Education, University of Perugia, Perugia, Italy; 5 Functional Neuroimaging Laboratory, IRCCS Santa Lucia Foundation, Rome, Italy

**Keywords:** Behavioural methods, Cognitive neuroscience, Attention

## Abstract

Cognitive flexibility involves the capability to switch between different perspectives and implement novel strategies upon changed circumstances. The Wisconsin Card Sorting Test (in humans) and the Attentional Set-Shifting Task (ASST, in rodents) evaluate individual capability to acquire a reward-associated rule and subsequently disregard it in favour of a new one. Both tasks entail consecutive stages wherein subjects discriminate between: two stimuli of a given category (simple discrimination, SD); the stimuli of SD confounded by an irrelevant stimulus of a different category (compound discrimination, CD); different stimuli belonging to the SD category (intradimensional shift, IDS); and two stimuli of the confounding category (extradimensional shift, EDS). The ASST is labour intensive, not sufficiently standardised, and prone to experimental error. Here, we tested the validity of a new, commercially available, automated version of ASST (OPERON) in two independent experiments conducted in: different mouse strains (C57BL/6 and CD1 mice) to confirm their differential cognitive capabilities (Experiment 1); and an experimental model of chronic stress (administration of corticosterone in the drinking water; Experiment 2). In both experiments, OPERON confirmed the findings obtained through the manual version. Just as in Experiment 1 both versions captured the deficit of C57BL/6 mice on the reversal of the CD (CDR), so also in Experiment 2 they provided analogous evidence that corticosterone treated mice have a remarkable impairment in the IDS. Thus, OPERON capitalises upon automated phenotyping to overcome the limitation of the manual version of the ASST while providing comparable results.

## Introduction

Executive functions (EFs) encompass a variety of heterogeneous cognitive processes involved in the control and monitoring of other—lower-level—cognitive functions^1^. The core components of EFs^2^ include the capability to inhibit behaviours and actions that are no longer appropriate to the purpose^3^; working memory, which is essential for the online maintenance of current goals^4,5^; and cognitive flexibility, i.e., the ability to vary and update effectively one’s goals and objectives^6^. EFs allow mammals to pay attention to relevant cues and disregard meaningless information, exert inhibitory control over actions, and take optimal decisions^7^. It is important to emphasize that these processes appear to be consistently supported by the frontal lobes, another important aspect to consider for enclosing these heterogeneous capabilities into a single set of control functions^8^. In particular, cognitive flexibility, which involves the capacity to rapidly switch between different perspectives and implement novel strategies to cope with adverse situations or changed circumstances^9^, is thought to be supported by the dorsolateral portions of the prefrontal cortex^10^. Lesions to this area produce severe deficits ascribed to the so-called dysexecutive syndrome^11^. A main feature of dysexecutive syndrome is the absence of cognitive flexibility, evidenced by the perseverative behaviour with which these patients continue to choose a previously learned rule, as revealed by the Wisconsin Card Sorting Test (WCST, e.g.^12^). The WCST, commonly used to investigate cognitive flexibility in humans^13^, consists of a set of cards—varying for geometric shapes, colours and numbers—that must be sorted by the participant according to categories that change periodically. In order to identify the correct category, participants need to adjust card sorting on the basis of the examiner’s positive or negative feedback (i.e., the chosen category is correct or incorrect, respectively), which follows any card sort. The WCST specifically highlights the inability of patients with frontal lobe impairments to inhibit the previously learned rule and to update it in favour of a new rule that would allow to achieve the intended purpose^14^. Just as the WCST, the Cambridge Neuropsychological Test Automated Battery Intra-/Extra-Dimensional set-shift task (CANTAB ID/ED task^15,16^) evaluates rule acquisition and rule update focusing on EFs and can be considered a computerized analogue of the WCST.

Mental disorders, be them due to brain injuries or neuropsychiatric pathologies, are often associated with impaired EFs (DSM-5^17^). While representing a diagnostic feature in major and mild neurocognitive disorder (e.g., due to Alzheimer's disease, traumatic brain injury, HIV infection, Huntington's disease and substance-use disorder), compromised EFs are also an associated symptom in attention-deficit/hyperactivity disorder (ADHD) and schizophrenia (DSM-5). These disorders entail behavioural, neuroanatomical and physiological symptoms, and molecular, genetic and environmental causative factors. To detail the fundamental mechanisms underlying multifactorial neuropsychiatric disorders, experimental animal models have traditionally constituted a tool of choice in biomedical sciences. Within this framework, several authors designed and developed experimental models characterised by alterations at the level of the brain structures involved in EFs^18,19^. These have taken the form of lesion studies in which selected brain regions have been experimentally ablated (e.g.^20,21^), genetic engineering approaches targeting relevant molecular pathways (e.g.^22,23^), and environmental manipulations aimed at altering specific neurotransmitters (e.g.^24,25^), to name a few. Whilst these approaches reliably target physiological and neurological mediators of EFs, the paradigms to investigate this higher-order brain function in rodents are still scarce. Therefore, devising off-the-shelf tools to quantify EFs in laboratory animals represents a desirable goal holding promise to expand the translational value of preclinical studies of mental disorders. With respect to EFs, the Attentional Set-Shifting Task (ASST), originally developed by Birrell and Brown^20^, represents a welcome endeavour, whereby it translates the WCST to rodents, thus allowing the study of inhibitory control and cognitive flexibility^26^.

Similar to the WCST, the ASST evaluates individual capability to acquire a rule in order to obtain a reward, and to subsequently disregard such rule in favour of a new one, once the learning criterion has been attained by trial and error. The similarities between the WCST and the ASST are numerous: the reward of the WCST (experimenter feedback) is replaced in rodents by a palatable food reward located within a bowl underneath a digging medium; the dimensions of the deck of cards (geometric shapes, colours and numbers) are translated into odour, digging medium, and texture of the bowls, three highly ethologically-relevant dimensions in rodents; both tasks entail several stages assessing behavioural flexibility and inhibitory control with variable levels of difficulty. For example, with respect to the latter, in the transition between the simple discrimination and the compound discrimination (see the “Methods” section) the experimental subjects have to acquire a rule and follow such rule while disregarding a new dimension which is included as a confound. Afterwards, when requested to perform the reversal discrimination (see the “Methods” section) they have to inhibit their acquired response in favour of a new one which was previously incorrect. Finally, in the extra-dimensional shift stage, mice display their cognitive flexibility.

Beside its face validity, this task has proven elevated construct (sensitivity to procedures aimed at altering the biological substrates involved) and predictive (sensitivity to pharmacological treatments) validities. With respect to the former, Birrel and Brown^20^ demonstrated that lesions of the frontal lobes in rats relate to deficits in the ASST; likewise, Hauser and co-authors^27^ demonstrated that exposure to neonatal milk devoid of specific nutrients involved in neuronal patterning results in impaired EFs in the ASST. With respect to the latter, while we recently observed that methylphenidate administration (a compound used to mitigate attentional deficits in ADHD patients) improved mouse performance in the ASST^28^, Bissonette and co-authors^18^ demonstrated that topiramate (a mood stabiliser acting on the GABAergic system) administration impaired the cognitive flexibility components of the ASST. Additionally, one of the key features of the ASST is represented by the fact that it leverages a series of stimuli and behavioural responses that are part of the rodent ethological repertoire.

Despite its numerous advantages, the ASST presents some core limitations. First, its execution is extremely labour intensive and requires the constant engagement of a highly-trained experimenter; second, the numerous variables involved (e.g., odours, digging media, rewards, positioning of the reward within the bowl) are likely to generate a considerable variation within and between laboratories; third, the combination of the aforementioned aspects increases the number of potential mistakes (e.g., inversion of rewarded odours); fourth, complete blinding of the experimenter is difficult to attain.

A potential solution to these limitations has been recently proposed through the design and development of a fully-automated version of the ASST^29^. This automated task closely mimics the WCST and the CANTAB ID/ED task, the two most widely used neuropsychological tests for the evaluation of attentional set-shifting abilities in humans. Recently, this version has been made commercially available (Operon 49500, Ugo Basile^®^ S.r.l., Gemonio, Italy^30^). The latter allows the execution of a modified yet analogous version of the ASST with minimal human supervision. Similar to the manual version, OPERON allows the completion of all the stages of the ASST using palatable food rewards delivered upon the execution of an operant response. OPERON also affords three dimensions (olfactory, tactile-visual, and visual) varying along several sets of stimuli as the cues to which experimental subjects have to pay attention throughout the test. Yet, these sets of stimuli are different from those offered by the manual version (in particular, for the tactile-visual dimension, texture of the conditioning area rather than digging medium). Likewise, the operant response, insertion of the nose into a nose-poke hole instead of digging, is radically different between the two versions. Ultimately, despite the theoretical similarities, the manual version of the ASST and its automated incarnation, present several discrepancies that we aim to fully unravel.

In the current study, we conducted a back-to-back comparison of the manual (ASST) and automated (OPERON) versions of this task to test whether they provide analogous results. To this aim, we approached this question through several converging avenues: first, we tested whether the two versions are equivalently sensitive to strain differences (Experiment 1); second, we assessed whether the effects of chronic stress—in the form of corticosterone (hereafter CORT) administration in the drinking water (see e.g.^31^)—on attentional set-shifting capabilities, previously reported to be sensitive to diverse stressors^32^, remain stable when tested through OPERON (Experiment 2). We thus tested, in both versions of the ASST task: (Experiment 1) independent cohorts of C57BL/6 and CD1 mice; and (Experiment 2) C57BL/6 administered CORT in the drinking water, and their controls (hereafter VEH).

## Results

**Figure 1:**
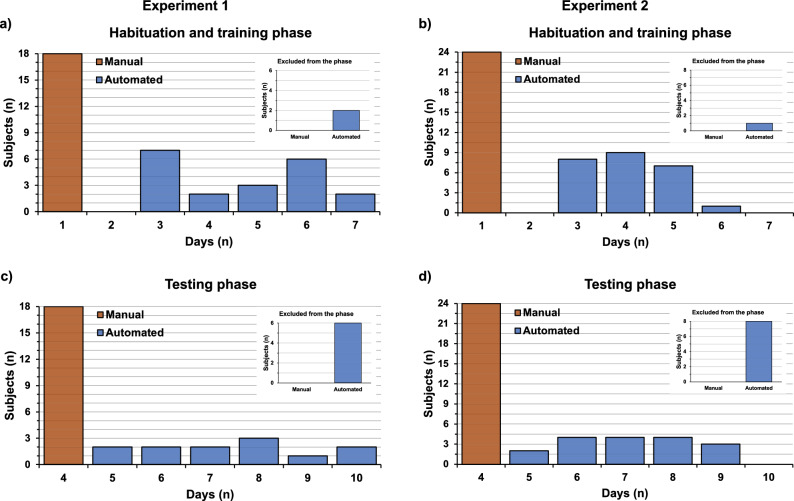
Number of subjects achieving the completion of the manual and automated version of the task on the specific day reported in the x-axis (main panels), or failing to complete the task (inset). (**a**) Number of mice tested in the manual (CD1: n = 9; C57: n = 9) and automated (CD1: n = 8; C57: n = 10) ASST in relation to the number of days employed to complete the habituation and training phase in Experiment 1. Inset: Mice that failed to complete the habituation and training phase of the automated ASST (CD1: n = 2; C57: n = 0). (**b**) Number of mice tested in the manual (VEH: n = 12; CORT: n = 12) and automated (VEH: n = 12; CORT: n = 13) ASST in relation to the number of days employed to complete the habituation and training phase in Experiment 2. Inset: Mice that failed to complete the habituation and training phase of the automated ASST (VEH: n = 1; CORT: n = 0). (**c**) Number of mice tested in the manual (CD1: n = 9; C57: n = 9) and automated (CD1: n = 6; C57: n = 6) ASST in relation to the number of days employed to complete the five stages of the testing phase in Experiment 1. Inset: Mice that failed to complete the testing phase of the automated ASST (CD1: n = 2, 1 in CDR and 1 in EDS; C57: n = 4, 1 in SD, 1 in CD, 1 in CDR, 1 in IDS). (**d**) Number of mice tested in the manual (VEH: n = 12; CORT: n = 12) and automated (VEH: n = 10; CORT: n = 7) ASST in relation to the number of days employed to complete the five stages of the testing phase in Experiment 2. Inset: Mice that failed to complete the testing phase of the automated ASST (VEH: n = 2, 1 in SD and 1 in CD; CORT: n = 6, 4 in SD, 1 in CD, 1 in CDR).

The manual testing phase was performed over 4 days whilst the automated testing phase required a minimum of five and a maximum of 10 days. The habituation and training phase lasted 1 day in the manual version and a minimum of three and a maximum of seven days in the automated version. The number of days required to complete the habituation and training phase and the five stages of testing, in the manual and automated versions, are shown in Fig. 1a–d.

Regarding Experiment 1, whilst all subjects (CD1: n = 9; C57: n = 9) tested in the manual version completed the task and were therefore included in the analyses, some animals (CD1: n = 4; C57: n = 4) did not complete the OPERON as they did not reach the criterion within the established number of sessions and could not continue the test. The Fisher’s exact test, performed to highlight within-strain differences in the number of animals that failed to complete the manual vs. automated ASST, did not reveal any significant differences (habituation and training phase CD1 P = 0.4737, C57 P = 1.000; testing CD1 P = 0.2059, C57 P = 0.0867).

In Experiment 2, all subjects tested in the manual version completed the task and were included in the analyses (VEH: n = 12; CORT: n = 12). Conversely, in the OPERON, some animals did not reach the criterion within the established number of sessions and were excluded from the test and the analyses (VEH: n = 3; CORT: n = 6). In the absence of within-group differences in the habituation and training phase (VEH P = 1.000, CORT P = 1.000), the Fisher’s exact test indicated that a higher number of CORT subjects failed to complete the automated task compared to the manual ASST (VEH P = 0.4783, CORT P = 0.0149).

### Separated analysis of manual and automated versions

#### Experiment 1

**Figure 2:**
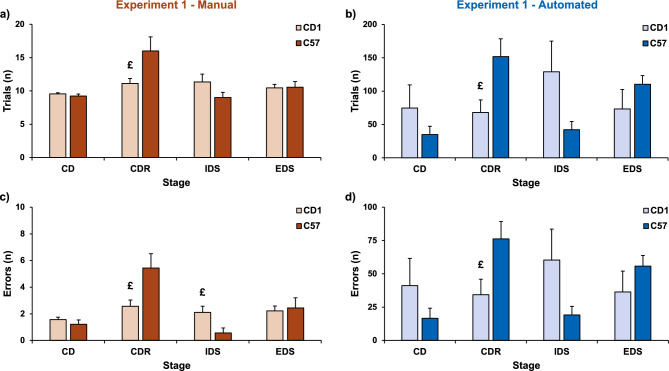
Trials to criterion and errors committed in the manual and automated version of the task in Experiment 1. (**a**) Mean number (± SEM) of trials necessary to complete each stage of the manual ASST (CD1: n = 9; C57: n = 9). (**b**) Mean number (± SEM) of trials necessary to complete each stage of the automated ASST (CD1: n = 6; C57: n = 6). (**c**) Mean number (± SEM) of errors committed to complete each stage of the manual ASST (CD1: n = 9; C57: n = 9). (**d**) Mean number (± SEM) of errors committed to complete each stage of the automated ASST (CD1: n = 6; C57: n = 6). ^£^P ≤ 0.05 compared to C57 mice in post-hoc test. For data regarding the first stage of the task, which required only a simple discrimination on one stimulus dimension, see Supplementary material, paragraph 2.1.1.

In the manual version, no differences were found between CD1 and C57BL/6 mice in the acquisition of the CD stage, which was characterised by the introduction of the irrelevant dimension (strain: F(1,16) = 0.82, P = 0.379 for trials, Fig. 2a; F(1,16) = 0.82, P = 0.379 for errors, Fig. 2c). A similar profile emerged in the CD stage of the automated version (strain: F(1,10) = 1.20, P = 0.298 for trials, Fig. 2b; F(1,10) = 1.25, P = 0.289 for errors, Fig. 2d). In the reversal of the CD stage (CDR), which required the formation of a new rule, we found a significant difference between the two strains, with decreased attentional performances in C57BL/6 compared to CD1 mice, in both the manual (strain: F(1,16) = 4.79, P = 0.044 for trials, Fig. 2a; F(1,16) = 6.10, P = 0.025 for errors, Fig. 2c) and the automated version (strain: F(1,10) = 6.49, P = 0.029 for trials, Fig. 2b; F(1,10) = 5.69, P = 0.038 for errors, Fig. 2d). Specifically, C57BL/6 mice required more trials and committed more errors than CD1 to attain the CDR, in both versions. No differences between the two strains were found in the number of trials to attain the IDS stage, either in the manual (strain: F(1,16) = 2.72, P = 0.118, Fig. 2a) or in the automated (strain: F(1,10) = 3.29, P = 0.100, Fig. 2b) version. However, CD1 mice committed more errors than C57BL/6 in the manual (strain: F(1,16) = 6.94, P = 0.018, Fig. 2c), but not in the automated (F(1,10) = 2.92, P = 0.118, Fig. 2d) version. In the manual version, no differences were found between CD1 and C57BL/6 mice in attaining the EDS stage, which required an attentional shift to the previously irrelevant dimension (strain: F(1,16) = 0.01, P = 0.913 for trials, Fig. 2a; F(1,16) = 0.07, P = 0.797 for errors; Fig. 2c). A similar profile emerged in the EDS stage of the automated version (strain: F(1,10) = 1.34, P = 0.274 for trials, Fig. 2b; F(1,10) = 1.20, P = 0.299 for errors, Fig. 2d).

#### Experiment 2

We confirmed that CORT administration significantly increased CORT concentrations in plasma. Thus, compared to VEH-treated controls, CORT-treated subjects had a substantial increase in plasma CORT concentrations (treatment: F(1,22) = 17.93, P < 0.001; CORT: 7748.30 ± 1695.54 pg/ml; VEH: 557.01 ± 99.81 pg/ml).

**Figure 3:**
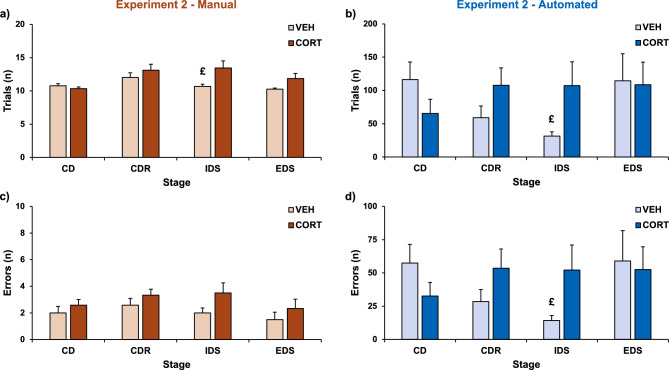
Trials to criterion and errors committed in the manual and automated version of the task in Experiment 2. (**a**) Mean number (± SEM) of trials necessary to complete each stage of the manual ASST (VEH: n = 12; CORT: n = 12). (**b**) Mean number (± SEM) of trials necessary to complete each stage of the automated ASST (VEH: n = 10; CORT: n = 7). (**c**) Mean number (± SEM) of errors committed to complete each stage of the manual ASST (VEH: n = 12; CORT: n = 12). (**d**) Mean number (± SEM) of errors committed to complete each stage of the automated ASST (VEH: n = 10; CORT: n = 7). ^£^P ≤ 0.05 compared to CORT mice in post-hoc test. Data regarding the first stage of the task, which required only a simple discrimination on one stimulus dimension, are reported in the Supplementary material, paragraph 2.1.2.

The treatment with CORT solution did not impair the performance of the mice in the CD stage both in manual (treatment: F(1,22) = 0.92, P = 0.348 for trials, Fig. 3a; F(1,22) = 0.85, P = 0.367 for errors, Fig. 3c) and in automated version (treatment: F(1,15) = 1.95, P = 0.183 for trials, Fig. 3b; F(1,15) = 1.71, P = 0.211 for errors, Fig. 3d). No differences between the two groups were found in the number of trials to attain the criterion or number of errors committed in the CDR stage in manual version (treatment: F(1,22) = 0.89, P = 0.357 for trials, Fig. 3a; F(1,22) = 1.25, P = 0.276 for errors, Fig. 3c). A similar pattern emerged in the CDR stage of the automated version (treatment: F(1,15) = 2.59, P = 0.129 for trials, Fig. 3b; F(1,15) = 2.44, P = 0.139 for errors, Fig. 3d). In the IDS stage, CORT administration affected the performance of the experimental subjects, with the CORT group showing decreased attentional capabilities both in manual (treatment: F(1,22) = 6.03, P = 0.022 for trials, Fig. 3a; F(1,22) = 3.13, P = 0.091 for errors, Fig. 3c) and in automated version (treatment: F(1,15) = 6.22, P = 0.025 for trials, Fig. 3b; F(1,15) = 5.46, P = 0.034 for errors, Fig. 3d). Then, in the manual version, no differences between control and CORT subjects were found in attaining the EDS stage (treatment: F(1,22) = 3.94, P = 0.06 for trials, Fig. 3a; F(1,22) = 0.85, P = 0.366 for errors, Fig. 3c). A similar profile emerged in the EDS stage of the automated version (treatment: F(1,15) = 0.01, P = 0.921 for trials, Fig. 3b; F(1,15) = 0.04, P = 0.84 for errors, Fig. 3d).

### Combined analysis of manual and automated versions

The measures obtained in the manual and automated versions were on different scales: in Experiment 1, while in the manual version, the average number of trials and errors per stage were respectively 10.9 (range 8–30) and 2.3 (range 0–12), in the automated version, these numbers were 85.5 (range 10–295) and 42.5 (range 2–144), respectively. Experiment 2 paralleled Experiment 1 whereby, in the manual version, the average number of trials and errors were 11.9 (range 10–21) and 2.7 (range 0–8), and in the automated they were 87 (range 10–419) and 42.7 (range 2–235), respectively. Therefore, to allow the direct comparison of these measures on different scales, we devised two distinct approaches: the standardisation based on the version, intended to minimise the effect of this variable; and the calculation of an additional variable called efficiency, focusing on the proportion of errors committed (see section Statistical analyses).

#### Experiment 1

##### Trials to criterion and errors committed following standardisation

As a result of the version-based standardisation, all effects involving the version were not significant (for trials, version: F(1,26) = 0.05, P = 0.832); strain × version: F(1,26) = 0.09, P = 0.768; stage × version: F(3,78) = 0.22, P = 0.880; stage × version × strain: F(3,78) = 0.30, P = 0.823; for errors, version: F(1,26) = 0.06, P = 0.813; strain × version: F(1,26) = 0.13, P = 0.723; stage × version: F(3,78) = 0.14; P = 0.932; stage × version × strain: F(3,78) = 0.20, P = 0.896).

**Figure 4:**
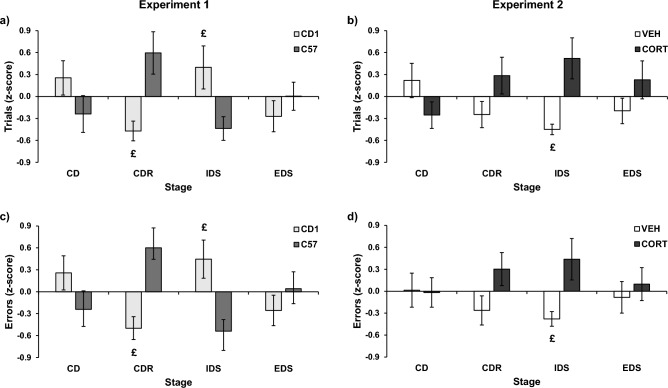
Trials to criterion and errors committed following a z-score normalization based on the version, intended to minimise the effect of this variable and to allow the direct comparison of measures on different scales. (**a**) Standardised mean number (± SEM) of trials necessary to complete each stage of the ASST in Experiment 1 (CD1: n = 15; C57: n = 15). (**b**) Standardised mean number (± SEM) of trials necessary to complete each stage of the ASST in Experiment 2 (VEH: n = 22; CORT: n = 19). (**c**) Standardised mean number (± SEM) of errors committed to complete each stage of the ASST in Experiment 1 (CD1: n = 15; C57: n = 15). (**d**) Standardised mean number (± SEM) of errors committed to complete each stage of the ASST in Experiment 2 (VEH: n = 22; CORT: n = 19). ^£^P ≤ 0.05 compared to C57 mice in (**a**) and (**c**) or to CORT mice in (**b**) and (**d**) in post-hoc test.

In the absence of main effects of strain (F(1,26) = 0.01, P = 0.940 for trials; F(1,26) = 0.002, P = 0.962, for errors) and stage (F(3,78) = 0.18, P = 0.910 for trials; F(3,78) = 0.13, P = 0.941, for errors), we found a significant stage by strain interaction for both the number of trials performed to reach the criterion and the number of errors committed (F(3,78) = 7.08; P < 0.001 and F(3,78) = 8.06, P < 0.001 respectively). This finding indicates that the differential profile of CD1 and C57BL/6 mice is maintained in both the manual and the automated task. Post-hoc analyses indicated that, compared to C57BL/6, CD1 mice performed significantly fewer trials in the CDR and significantly more trials in the IDS (Fig. 4a). A similar pattern was observed for errors (Fig. 4b).

##### Efficiency

To evaluate whether the two versions had a differential impact on the proportion of errors committed, we calculated the efficiency to complete each stage, calculated as [Trials performed to reach the criterion/(Trials performed to reach the criterion + Errors committed)] × 100. This approach allowed a direct comparison of the manual and automated versions.

**Figure 5:**
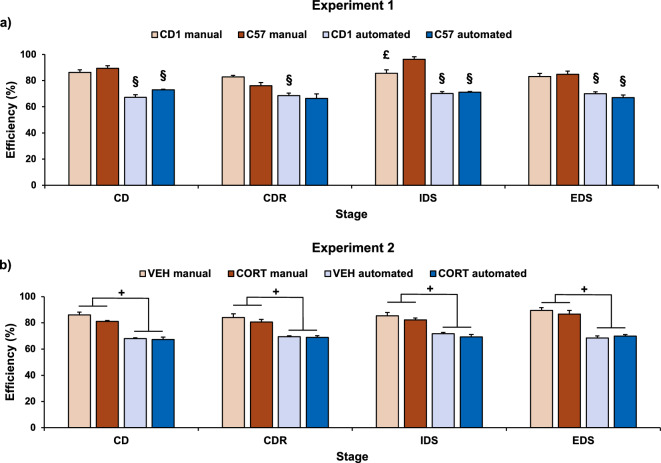
Efficiency, intended to evaluate whether the two versions had a differential impact on the proportion of errors committed. (**a**) Mean efficiency (± SEM) to complete each stage of the manual and automated version of the ASST in Experiment 1 (manual: CD1: n = 9; C57: n = 9; automated: CD1: n = 6; C57: n = 6). (**b**) Mean efficiency (± SEM) to complete each stage of the manual and automated version of the ASST in Experiment 2 (manual: VEH: n = 12; CORT: n = 12; automated: VEH: n = 10; CORT: n = 7). ^£^P ≤ 0.05 compared to C57 mice; ^§^P ≤ 0.05 compared to the corresponding manual version in post-hoc test; ^+^P ≤ 0.05 main effect of the version.

In Experiment 1, we found a main effect of the version (F(1,26) = 191.74, P < 0.001), with the manual task being on average significantly more efficient than the automated one. The efficiency varied from stage to stage (F(3,78) = 6.87, P < 0.001) depending on both the version and the strain (stage × version: F(3,78) = 2.11, P = 0.106; stage × strain: F(3,78) = 3.72; P = 0.015; stage × version × strain: F(3,78) = 1.80, P = 0.154). Specifically, CD1 mice performed all stages of the manual task with a significantly higher level of efficiency compared to the corresponding stages of the automated task (Fig. 5a). A similar profile was obtained for C57BL/6 mice except for the CDR stage, in which no differences of efficiency were found between the manual and the automated version. Finally, C57BL/6 mice performed the IDS stage of the manual task with a significantly higher level of efficiency compared to CD1 mice (Fig. 5a). The main effect of the strain and the strain by version interaction were not significant (F(1,26) = 1.22, P = 0.280; F(1,26) = 0.53, P = 0.475, respectively).

#### Experiment 2

##### Trials to criterion and errors committed following standardisation

As expected, all effects involving the version were not significant (for trials, version: F(1,37) = 0.03, P = 0.863); treatment × version: F(1,37) = 0.29, P = 0.592; stage × version: F(3,111) = 0.05, P = 0.986; stage × version × treatment: F(3,111) = 0.68, P = 0.564; for errors, version: F(1,37) = 0.02, P = 0.88; treatment × version: F(1,37) = 0.55, P = 0.462; stage × version: F(3,111) = 0.04; P = 0.988; stage × version × treatment: F(3,111) = 1.02, P = 0.388). In the absence of the main effect of the stage (stage: F(3,111) = 0.05, P = 0.99 for trials and F(3,111) = 0.04, P = 0.99 for errors), the minimisation of the effect of the version allowed us to show that CORT treatment affected the performance of experimental subjects both in the manual and in the automated task. Specifically, the CORT group showed reduced attentional performance compared to control subjects, requiring a higher number of trials and errors to complete the task (treatment: F(1,27) = 6.29, P = 0.017 for trials, Fig. 4c; F(1,27) = 6.02, P = 0.018 for errors, Fig. 4d). We found a significant stage by treatment interaction for trials performed to reach the criterion, with post-hoc analyses indicating that, compared to control subjects, the CORT group performed significantly more trials and committed more errors to complete the IDS stage (stage × treatment: F(3,111) = 3.97, P = 0.001 for trials, Fig. 4c; F(3,111) = 1.92, P = 0.131 for errors, Fig. 4d).

##### Efficiency

Both VEH and CORT subjects performed all stages of the manual task with a significantly higher level of efficiency compared to the corresponding stages of the automated task, with analyses showing a significant main effect of the version (F(1,37) = 167.365, P < 0.001), regardless of treatment (F(1,37) = 2.86, P = 0.099). Besides, the efficiency did not vary depending on stage or on interaction between different factors (treatment × version: F(1,37) = 1.7, P = 0.201; stage: F(3,111) = 1.37, P = 0.255; stage × treatment: F(3,111) = 0.17, P = 0.914; stage × version: F(3,111) = 1.37, P = 0.276; stage × treatment × version: F(3,111) = 0.13, P = 0.942, Fig. 5b).

## Discussion

The core aim of our study was to challenge the validity of a newly-developed, commercially available, automated version of the ASST (Operon 49500, Ugo Basile^®^ S.r.l., Gemonio, Italy^30^). To this aim, we stress-tested OPERON adopting two converging experimental strategies. On the one hand, we conducted the ASST using the traditional version and its automated incarnation in C57BL/6 and CD1 male mice; on the other hand, we evaluated whether OPERON was sufficiently sensitive to capture the differential cognitive capabilities observed in an experimental model of chronic stress. We decided to validate the automated version of the ASST only in males, since previous efforts reporting strain differences in its manual version have been conducted predominantly in males. While this does not provide a valid justification for continuing to conduct these studies exclusively in males, we opted for this decision since our goal was to confirm the effectiveness of the new test in an experimental group for which the anticipated differences had already been documented^33–35^.

We note that this is the first instance in which an automated version of the ASST is directly compared to the manual task.

In support of the validity of OPERON, we observed that C57BL/6 mice encountered more difficulties (i.e., required more trials) than CD1 mice in the CDR, be it conducted using the manual or the automated version. Likewise, both OPERON and the manual version were sufficiently sensitive to substantiate our prediction that mice exposed to a chronic stress procedure had impaired cognitive capabilities.

Previous studies investigated strain differences in this task and consistently reported major inter-individual variations. For example, in accordance with our data, Colacicco and co-authors^33^ observed that C57BL/6J mice exhibited impairments in selected stages of the ASST, albeit their control subjects were constituted by the inbred 129/SvEv strain and not by the outbred CD1 strain adopted herein. Accordingly, we previously reported that C57BL/6 exhibited significant impairments in the ASST task compared to ABH mice^34^. Strain differences in attentional capabilities between C57BL/6J and CD1 have also been reported in other behavioural paradigms. Oliver and co-authors^35^ observed that C57BL/6J mice showed impaired attentional capabilities compared to CD1 mice in the five-choice serial reaction time task (5-CSRTT). The latter measures the ability to focus the attention on a specific information, disregarding other stimuli^36^. It translates to rats and mice the continuous performance tests used to study attention in human^37,38^, thus allowing the evaluation of attention and impulsivity^39^. Strain differences between C57BL/6J mice and CD1 have also been reported in working memory, another core component of EFs^4,5^. Specifically, Sultana and co-authors^40^ reported that C57BL/6J mice exhibited decreased working memory compared to CD1 in the Y-maze test. These findings suggest that the impairment observed in this task in C57BL/6 mice has a remarkable degree of external validity whereby it has been reproduced in independent facilities adopting independent test strategies.

The capability of OPERON to replicate the differences observed in the manual version in two mouse strains extended to the disease model tested herein. Thus, mice chronically exposed to CORT exhibited significant impairments in IDS regardless of the ASST version adopted.

The ASST has been extensively used to reveal impairments in executive functions in several experimental models of chronic stress (for a review see^32^). With respect to CORT administration, Wallace and collaborators^41^ adopted an experimental strategy analogous to that adopted herein, and, in agreement with our findings, also reported that CORT-treated subjects exhibited impaired rule abstraction (IDS). Additionally, Nikiforuk and Popik further confirmed the role of CORT in mediating attentional capabilities in experimental models of chronic stress^42^. Thus, they first observed that chronically stressed rats had impairments in the ASST, and then demonstrated that the inhibition of CORT synthesis had a protective effect whereby it abolished the reported impairment^42^. The relevance of CORT as a candidate regulator of executive functions is further supported by our study. Thus, beside the impairment in IDS, we confirmed that CORT administration successfully increased plasma CORT concentrations.

With respect to the heuristic value of the ASST, the two experiments we conducted captured between-group differences in differential stages of the task. Thus, while we observed that CD1 and C57BL/6 mice differ in the CDR stage (linked to the orbitofrontal cortex^21^), CORT and control mice differ in the IDS stage (linked to the cingulate cortex^43^). The fact that these findings were consistent regardless of the level of task automation further strengthens: (i) the sensitivity of the ASST; and (ii) the remarkable correspondence between the manual and automated versions of the task.

With respect to the plasticity and flexibility of the automated version of the ASST, we note that the OPERON software can be easily customized to manage the experimental stages. Specifically, the protocol can be adjusted relatively seamlessly to meet specific experimental requirements. For example, just as the original manual version of the ASST entailed three different perceptual dimensions (^20^in rats; ^44^in mice), so also OPERON affords three dimensions. Yet, several authors reduced the number of required dimensions to two^18,25,27,33,34,45^. Importantly, using two dimensions, the EDS allows to test the inability to re-engage attention towards a previously irrelevant dimension (i.e., “learned irrelevance”), whilst with three dimensions it is possible to use the EDS to test for the inability to release attention from a relevant perception dimension (i.e., perseveration or “stuck-in-set”^30,46^). Such downscaling can be, and has been herein, easily attained in OPERON. Likewise, the number of stages adopted in literature varies between experiments. To account for this, OPERON offers a basic series of nine stages (as in the CANTAB), which can be easily reduced to meet specific experimental needs (e.g., SD, CD, CDR, IDS, EDS as in^27,28,34^).

The flexibility of OPERON allowed us to implement some task modifications that promoted the procedural and theoretical similarity with the manual version. Our modifications aimed at replicating in OPERON the performance-based nature of the manual version. Specifically, in the manual version, mice are allowed to continue performing the task as long as they are motivated to. Conversely, in the original version of OPERON^30^, mice were tested on a fixed schedule that would end upon attainment of a predetermined number of trials. We believe that, beside promoting the similarity between the two versions of the task, this expedient has the advantage of meeting individual needs and predispositions.

In the comparison of the two tasks, we observed that the efficiency of the manual version is remarkably higher than that of the automated one. Thus, the ratio between errors and trials to criterion is much lower in the traditional version, indicating that mice learn to discriminate the incorrect from the correct stimulus at a much faster pace. This result is likely related to the fact that the manual version has a much higher ethological validity compared to OPERON, whereby it rests upon a species-specific highly meaningful behavioural response (digging). This feature translates into the fact that the manual ASST barely requires any training and entails only a few days of testing. Whilst this aspect certainly results in a quicker completion of the task, it nonetheless may trigger potential floor effects, ultimately hampering the observation of subtle between-group differences.

One last aspect that warrants consideration relates to the number of subjects used in both versions of the task. In the present study, while all subjects tested in the manual version completed the task, approximately 40% of subjects tested in OPERON had to be discarded since they failed to reach the criterion within the established number of sessions. It is important to emphasize that a certain experimental dropout is the norm rather than an exception in operant tasks, e.g.^47^. We note, however, that the reported dropout did not hinder the quality of the observed findings. Albeit reduced in number, the animals tested in the automated version yielded experimental data that were analogous or even better in quality to those afforded by mice tested in the manual task. Specifically, C57BL/6 mice tested in the automated version required a higher number of trials to attain the EDS compared to the IDS. While this phenotype was predicted based on available literature (e.g.^43^), it was not observed in the manual version. Interestingly, similar patterns of performance were reported in humans, monkeys, rats, and mice, with initial difficulties in achieving the CDR and improved performance in the IDS compared with EDS^20,22,29,44,47,48^.

Ultimately, the detailed back-to-back comparison conducted in this study allows precise considerations regarding methodological and practical aspects potentially guiding scientists interested in the ASST. From a purely methodological perspective, compared to the manual version, OPERON requires a much lower level of training in behavioural neuroscience and affords a much higher degree of standardisation and automation. The latter also protects from potential mistakes (e.g., inversion of rewarded stimuli), which may occur in the light of the labour-intensive nature of the task and its associated tiredness. Additionally, OPERON guarantees by definition full experimenter blinding whereby it is fully automated and does not allow any room for the experimenter to unconsciously influence the response of the tested individual. For example, in our study, blinding was not possible since the experimental groups differed for the coat colour. In spite of these advantages, OPERON does not perform on par with the manual version concerning other relevant aspects. For example, the behavioural response required to obtain the reward in the manual version is much more ethologically relevant than that required in OPERON; this likely translates in the higher efficiency observed in the manual version, and in its largely reduced duration (two- to three-fold) compared to OPERON. We cannot exclude that this aspect also contributes to the lack of dropouts observed in the manual ASST. Finally, the economic dimension of the two versions of the ASST warrants consideration. While the initial investment is certainly in favour of the manual version (few hundred euros versus several thousands), an overall budget estimation cannot neglect the human costs associated with the conduction of this test. While the manual version requires a full-time commitment of a highly trained operator throughout the entire study, OPERON demands very minimal experimental supervision.

In conclusion, we show that, despite minor caveats, the automated version of the ASST bestows results analogous to those afforded by the manual version, while limiting manpower and sources of confound.

## Methods

### Ethics statement

All experimental procedures were approved by Institutional Animal Survey Board on behalf of the Italian Ministry of Health (licence n. 729/2020-PR to SM) and performed in full accordance with the Directive 2010/63/EU on the protection of animals used for scientific purposes and Italian law (Legislative Decree 26/2014). All sections of this report adhere to the ARRIVE Guidelines for reporting animal research. A completed ARRIVE Essential 10 Checklist is included as supplemental material.

### Animals and experimental design

#### Experiment 1

Experimental subjects were 19 adult male C57BL/6N mice and 19 adult male CD1 mice provided by Charles River Laboratories s.r.l. (Calco, Italy). Upon arrival C57BL/6N and CD1 mice, weighting approx. 20 g and 24 g respectively, were housed in pairs in polycarbonate cages (33.0 × 13.0 × 14.0 cm) equipped with metal tops. All animals had access to sawdust bedding, environmental enrichment in the form of shelter material (Nestlets^®^), and ad libitum water and food (Mucedola s.r.l., Settimo Milanese, Italy) until the beginning of the experiment (for details on the food restriction schedule see next paragraph). Animals were identified through ear-clipping and housed in an air-conditioned room (temperature 24 ± 1 °C, relative humidity 40 ± 5%), on a 12-h reversed light–dark cycle (lights on at 20:00). Within each strain, mice were randomly assigned to either the manual or the automated version of the ASST. Mice were tested daily, 7 days a week (except for the habituation and training phase of the automated ASST with 6 days of testing per week), under dim-light conditions. Behavioural tests were performed in an experimental room adjacent to the housing room, minimising gradients in light, temperature, sound, and other environmental conditions. To minimise potential confounders, all experimental subjects were housed in the same room; additionally, the position of the cages within the rack was randomly assigned. Animals were brought to the experimental room inside their home-cages, which were covered with a thick cloth. Testing started around 09:00 (i.e., 1 h after the light–dark shift) and ended no later than 19:00.

Regarding group allocation, conduct of the experiment, and outcome assessment, in the manual version blinding was not possible since (i) the experimental groups differed for the coat colour and (ii) data were manually scored by the experimenter. By contrast, the automated version guaranteed full experimenter blinding in the conduct of the experiment and outcome assessment.

#### Experiment 2

Experimental subjects were 50 adult male C57BL/6JOlaHsd (approx. 20 g), provided by ENVIGO RMS s.r.l. (San Pietro al Natisone, Italy). Upon arrival, mice were housed as described in Experiment 1. After 10 days of habituation, half of the mice were randomly assigned to a CORT treatment while the other half to VEH using online random number generator. CORT (Sigma-Aldrich, Missouri, USA) treated mice received CORT dissolved in their drinking water at a dose of 0.035 mg/ml, starting from 3 weeks before the beginning and throughout the duration of the experiment. The single mouse constituted the experimental unit.

The average liquid intake was 5.78 ± 0.16 ml per day. In consequence of the ad libitum access to the CORT solution, mice received on average 8.60 ± 0.33 mg/kg/day of CORT. Details regarding the preparation of the CORT and VEH solutions are reported in the Supplementary material (paragraph 4.2.2.). Testing of mice was performed as described in Experiment 1. At the end of the experiment (after 8 weeks of CORT treatment), blood samples were collected from CORT (n = 12) and VEH (n = 12) mice tested in the manual ASST to evaluate basal CORT concentrations in plasma (see Supplementary material, paragraph 4.2.2., for details).

For the manual version, as already mentioned, in the conduct of the experiment and outcome assessment blinding was not possible since. By contrast, the automated version guaranteed full experimenter blinding in the conduct of the experiment and outcome assessment. In addition, for both versions, the experimenter was not aware of group allocation.

### Food restriction schedule

In both Experiment 1 and 2, the food restriction, applied to increase animals’ motivation to perform the task, aimed at maintaining the animals at 85–90% of their free feeding bodyweight. Before food restriction, the average body weight of experimental subjects in Experiment 1 was: 25.8 ± 0.4 (C57BL/6N) and 30.2 ± 0.5 (CD1). The average body weight of experimental subjects in Experiment 2 was: 26.7 ± 0.6 (VEH) and 26.4 ± 0.4 (CORT). Food restriction was applied, 7 days a week by giving mice access to food for a limited time after completing the daily session. Specifically, mice were daily weighed and the time available for access to food (usually in the range between 2 and 4 h) was individually adjusted in order to allow the subject’s bodyweight to remain constant. To this aim, the order of testing was daily scheduled based on bodyweight percentages (from the lower to the higher), also to guarantee an extended access to food (e.g. 4 h) when needed. Mice were also daily inspected to identify potential signs of poor wellbeing (e.g., locomotion, fur condition). Mice were food-restricted from 3 days before the beginning of the habituation and training phase and throughout the testing phase.

### Manual version of the attentional set-shifting task (ASST)

**Figure 6:**
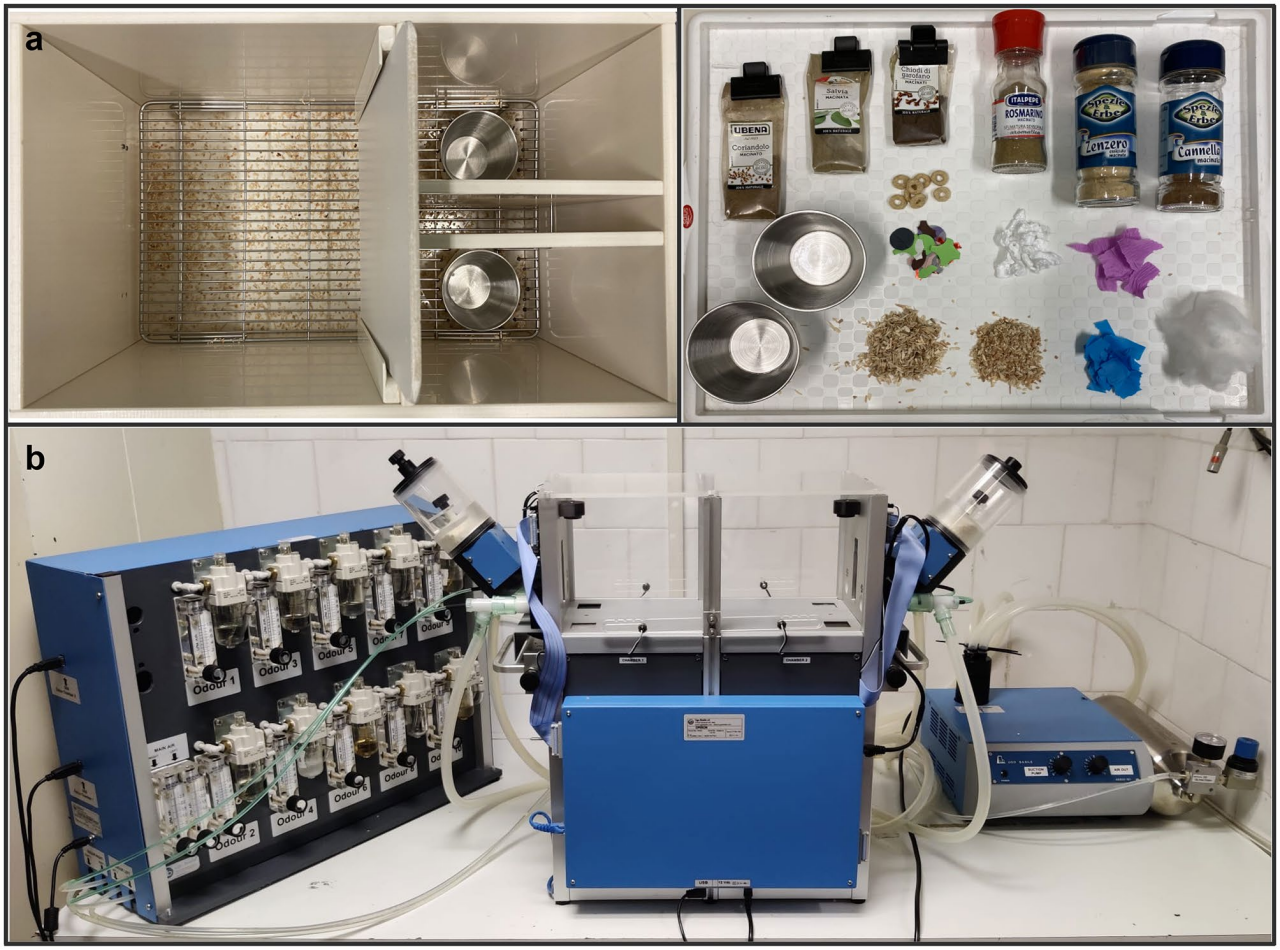
Setting for the manual and automated attentional set-shifting task (ASST) in mice. (**a**) Photographs of the apparatus for the manual task taken from above (on the left) and of odours and digging media used as stimuli (on the right). (**b**) Photograph of the apparatus (Operon 49500, Ugo Basile^®^ S.r.l., Gemonio, Italy) for the automated task (in the middle), with the odour-delivery unit (on the left) and the pump for the delivery/aspiration system (on the right). In manual ASST, we mixed small amounts of powdered aromatic herbs/spices with the digging media. In automated ASTT, odours were prepared by diluting liquid flavours employed in the confectionery industry (5% in mineral oil).

In both Experiment 1 and 2, the manual test was conducted in a custom-made opaque PVC U-maze (45 × 30 × 15 cm) with one starting compartment (30 × 30 cm) connected, through a sliding door, to two identical choice compartments (15 × 15 cm; Fig. 6a). A metal bowl (4.0 cm high, 7.0 cm top diameter, 4.0 cm bottom diameter) was placed in each of the two identical compartments and a small piece of cereal (1/4 honey Cheerios^®^, Nestlè, Vevey, Switzerland), covered with 2.0 cm layer of digging medium, was used as a reward (as in^26^). Under the wire-mesh floor of the maze we placed a layer of sawdust bedding from the home-cage of the tested subject to provide a more familiar environment and to reduce the stress due to exposure to a novel environment. Between sessions, the apparatus was cleaned with 30% ethanol/water solution to remove odour cues.

#### Habituation and training phase

The training phase was performed on the first day and consisted of nine trials, preceded by 5 min of habituation to the apparatus. This phase aimed at training mice to reliably dig into the bowls to obtain the reward. During habituation, the sliding door was removed, both bowls were empty, and mice were free to explore the whole apparatus. During training, each trial started with the mouse positioned in the starting compartment and the sliding door closed. When the mouse was facing the wall of the apparatus opposite to the door, the latter was manually raised by the experimenter, allowing contemporary access to both choice compartments. During training, both bowls were baited, and mice had to retrieve and consume both rewards to complete the trial. When the mouse spontaneously returned to the starting compartment after having retrieved the second reward, the trial was terminated by closing the sliding door. The nine trials of the training phase were arranged as follows: rewards placed at the bottom of the empty bowls in trials 1–3; rewards placed on top of the bowls filled with the same type of sawdust used as bedding material in trials 4–6; rewards covered by a 2.0 cm layer of bedding material in trials 7–9.

#### Testing phase

**Figure 7:**
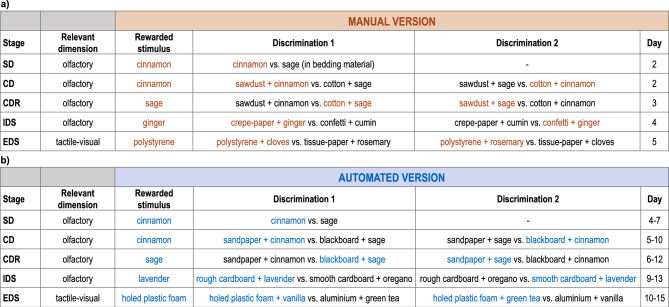
Schematic representation of the five stages of the (**a**) manual and (**b**) automated ASST testing phase (first column), with the dimension that was relevant (second column) and the stimulus that was rewarded (third and seventh columns) in each stage (brown and blue are used for the manual and automated versions respectively). In each stage, mice were presented with either Discrimination 1 (fourth and eighth column) or Discrimination 2 (fifth and ninth columns), according to a pseudo-random sequence. The side where each pair of stimuli was presented (left and right bowls in the manual task, left and right nose-poke holes in the automated task) were counterbalanced through a pseudo-random sequence. In the sixth and tenth columns we reported the range of days in which each stage of the task was completed.

The testing phase consisted of five consecutive stages, with different discriminations involving stimuli belonging to two dimensions (i.e., olfactory and tactile-visual). The stimuli presented in each stage (i.e., odours and digging media) are shown in Fig. 7a. In the first stage (simple discrimination; SD), the animal was presented with only the olfactory dimension, having to perform a simple discrimination between two different stimuli (i.e., two odours). In the second stage (compound discrimination; CD), we introduced the tactile-visual dimension with two additional stimuli (i.e., two digging media); however, the relevant dimension (i.e., olfactory dimension) and the rewarded stimulus remained consistent with the SD. In the third stage (compound discrimination reversal; CDR), the four stimuli were maintained from the CD; the irrelevant dimension in the previous stage (i.e., tactile-visual dimension) continued to be irrelevant but the rewarded stimulus was now the one that had been previously incorrect. For the fourth (intra-dimensional shift; IDS) and fifth (extra-dimensional shift; EDS) stages, we introduced four novel stimuli in both dimensions (i.e., two odours and two digging media). Whilst in the IDS the relevant dimension remained the olfactory one, in the EDS subjects had to shift their attention to the previously irrelevant dimension (i.e., tactile-visual dimension).

At the beginning of each testing trial the mouse was positioned in the starting compartment and the sliding door was closed. As soon as the mouse happened to be far from the door and facing the opposite wall, the experimenter manually raised the sliding door to give the subject access to the two choice compartments. During testing, only one bowl was baited. Choice was defined as the first evident digging in either the baited or the unbaited bowl. Each stage began with four exploratory trials^20,49^ during which the door remained open even if the first choice was incorrect, and the mouse was allowed to investigate and collect the reward from the opposite bowl. The outcome of these initial trials was included in the presented results (as in^27^). In subsequent trials, (i) if the subject started to dig in the unbaited bowl, an error was recorded, and the trial was terminated by closing the door and leaving the mouse within the choice compartment for 1 min (punishing timeout); (ii) if the subject started to dig in the baited bowl, the trial was terminated by closing the door and allowing the mouse 1 min to consume the reward within the choice compartment. In both correct and incorrect trials, after the 1-min timeout, the sliding door was opened, and the subject spontaneously returned to the starting compartment.

In order to complete each stage, mice had to reach a criterion of eight correct discriminations out of ten consecutive trials. Each day testing continued until the mouse was willing to work (usually about 60 min); specifically, a 10-min cutoff was set for each trial, allowing the experimenter to terminate testing when the mouse did not express any choice in the 10 min following the beginning of a trial.

### Automated version of the attentional set-shifting task (ASST)

The test was conducted using the upgraded version of the OPERON task, originally developed by Scheggia and Papaleo^29,50^, that is now commercially available (Operon 49500, Ugo Basile^®^ S.r.l., Gemonio, Italy). For a thorough description of the apparatus, the reader is referred to Scarsi and co-authors^30^, where the commercially available version was firstly used.

Briefly, the apparatus consisted of a double-chambered cage, with transparent walls and metal floor, divided by an automated metal sliding door placed in the middle of the cage, and with an operant stimulation wall mounted on each side (Fig. 6b).

**Figure 8:**
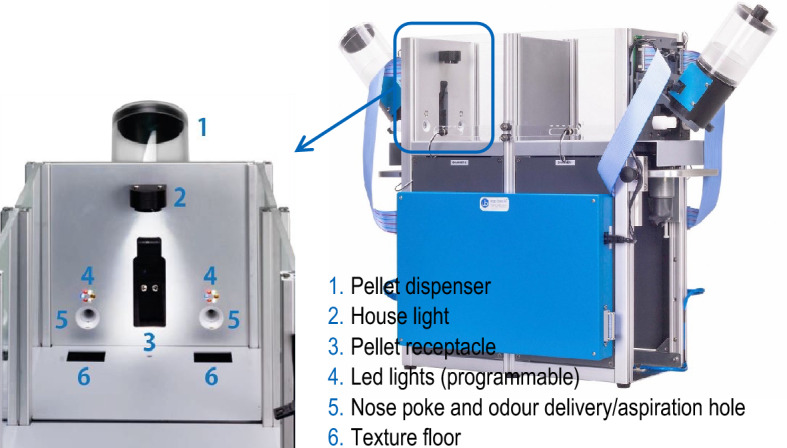
Operon apparatus. On the right, side view of the double-chambered cage (Operon 49500, Ugo Basile^®^ S.r.l., Gemonio, Italy), divided by the central sliding door, with an operant stimulation wall mounted on each side. On the left, particular of the stimulation wall (front view) highlighting the position of the following elements: external pellet dispenser (**1**); house light placed in the top middle of the wall, signalling the length of the punishing timeout (**2**); central pellet receptacle where precision pellets were delivered (**3**); programmable arrays of led lights, providing the stimuli belonging to the visual dimension, placed over each nose-poke hole (**4**); nose-poke holes equipped with the odour delivery/aspiration system, providing the stimuli belonging to the olfactory dimension (**5**); texture floors, providing the stimuli belonging to the tactile-visual dimension, placed under each nose-poke hole (**6**).

Each operant wall included automated stimulators involving stimuli belonging to three dimensions (i.e., olfactory, tactile-visual, visual). Specifically, each stimulation wall was equipped with an external pellet dispenser, a central pellet receptacle where precision pellets were delivered (when the correct choice was made), a single house light placed in the top middle of the wall (signalling the length of the punishing timeout when the incorrect choice was made), two nose-poke holes (one of each side of the pellet receptacle), two programmable arrays of coloured led lights (providing the stimuli belonging to the visual dimension) placed over each nose-poke hole, and two texture floors (providing the stimuli belonging to the tactile-visual dimension) placed under each nose-poke hole (Fig. 8). The latter were part of the revolving floor system for automated tactile stimulation, which was mounted under the floor in front of each stimulation wall.

The nose-poke holes were equipped with an odour delivery/aspiration system (providing the stimuli belonging to the olfactory dimension) and connected to the odour-delivery unit, which provided automated odour stimulation via different odours (up to ten) independently in the two chambers. The odour-delivery unit was composed of ten flasks (one for each odour) with individual pressure control. Importantly, the delivery/aspiration system allowed to both deliver the odour inside the nose-poke hole and to extract the air from it after the stimulation, cleaning the hole from the odour previously used before the initiation of the subsequent trial.

The setting allowed the subject to interact with the pair of stimuli (i.e., odours and textures; the visual dimension, involving the coloured led lights, was not used in the present experiment) presented on the two sides of each operant wall, and to express its choice by inserting the nose in one of the two nose-poke holes. A 14 mg non-flavoured precision pellet (5TUL purified rodent tablets, catalog n. 1811215; TestDiet, St. Louis, MO 63144, USA), delivered in the pellet receptacle, was used as a reward.

The two chambers allowed for continuous trial repetition: at the beginning of the session the mouse was confined by the sliding door in the first chamber; after having interacted with the stimulation wall inside this chamber, the sliding door lowered, and the mouse shuttled to the opposite chamber. After having expressed its choice between the two nose-poke holes of the stimulation wall inside the second chamber, the mouse shuttled back to the first chamber where new pairs of stimuli (i.e., odours and textures) had been automatically arranged meanwhile. During both the training and the testing phase, the total number of trials that could be performed within each session was not predetermined, as mice were free to express nose-poking at their own, individually variable rate.

Infrared photo-beams, placed inside the nose-poke holes, the pellet receptacle and in proximity to the sliding door, were used to detect the activity of the animals (i.e., nose-poking, eating the rewards, shuttling to and fro). Following the delivery of the reward from the pellet dispenser, only the photo-beam inside the pellet receptacle was active. Hence, additional nose-poking was without any consequences until the interruption of the pellet receptacle photo-beam. After the reward had been eaten (in correct trials) or after the 5-s punishing timeout (in incorrect trials), the sliding door lowered, and the subject spontaneously moved to the opposite chamber to start the next trial. As soon as the photo-beam of the new chamber was interrupted, the sliding door automatically rose, confining the mouse in this chamber.

A dedicated version of the ANY-maze software (Stoelting Co., Wood Dale, IL, USA) automatically managed all OPERON cues, tools and sensors following a precompiled protocol and collected the data. Between sessions, the apparatus was cleaned with 30% ethanol/water solution to remove odour cues.

#### Habituation and training phase

The habituation was conducted on the first 2 days and consisted of two parts, each having a fixed duration of 40 min.

During the first part of the habituation, performed on the first day, lights were turned off, no odours were delivered inside the nose-poke holes, the texture floors were neutral (i.e., made of the same material of the apparatus floor), and the sliding door remained lowered. Inserting the nose in any of the four available nose-poke holes resulted in the delivery of the reward in the pellet receptacle of the corresponding operant wall. Until the interruption of the pellet receptacle photo-beam, additional nose-poking did not result in the delivery of additional pellets.

The second part of the habituation, performed on the second day, was similar to the first one, except for the sliding door that was activated. At the beginning of this second part of the habituation, the animal was confined by the sliding door in the first chamber, the door was then automatically lowered, allowing the animal to move to the second chamber, thus starting the first trial; as soon as the animal had entered this chamber, the sliding door automatically rose. Nose-poking in either of the two holes of the operant wall within the chamber resulted in the delivery of the reward in the pellet receptacle. Following the interruption of the pellet receptacle photo-beam, the sliding door lowered, and the animal had to move back to the first chamber to start the subsequent trial.

The training phase was conducted from the third day onwards and consisted of a variable number of daily sessions (up to five) depending on the animal performance (see below). Each daily session had a maximum duration of 40 min. In this phase, the animal was presented with the olfactory dimension, having to perform a simple discrimination between two different stimuli (i.e., two odours). Hence, only one of the two nose-poke holes was associated with the reward. The correct stimulus was randomly presented in either the right or the left nose-poke hole of the operant wall. The training phase began with ten exploratory trials during which the mouse was allowed to nose-poke in the other hole and collect the reward even if the first choice was incorrect. In subsequent trials, (i) if the subject nose-poked in the incorrect hole, an error was recorded and the trial was terminated by turning on the house light to signal the punishing timeout whilst all photo-beams were inactivated; (ii) if the subject nose-poked in the correct hole, the reward was delivered in the pellet receptacle. After the 5-s punishing timeout (in incorrect trials) or after the reward had been eaten (in correct trials), the sliding door lowered, and the subject spontaneously moved to the opposite chamber to start the next trial. As soon as the photo-beam of the new chamber was interrupted, the sliding door automatically rose confining the mouse in this chamber. In order to complete the training phase, mice had to reach a criterion of eight correct discriminations out of ten consecutive trials (the ten exploratory trials were included in the scoring). Animals that failed to reach this criterion during the available five daily sessions were excluded from the testing phase.

#### Testing phase

As in the manual version of the task, the testing phase consisted of five consecutive stages, with different discriminations involving stimuli belonging to two dimensions (i.e., olfactory and tactile-visual). The stimuli (i.e., odours and texture floors) presented in each stage are shown in Fig. 7b. For a description of the five stages the reader is referred to the corresponding paragraph in the section “Manual version of the attentional set-shifting task (ASST)”, as the only difference was the use of texture floors in place of the digging media. Each stage of the testing phase consisted of a variable number of daily sessions (up to three for SD, CD and IDS, and up to five for CDR and EDS) depending on the animal performance.

As for the functioning of the apparatus (e.g., nose-poke holes, sliding door, photo-beams, house light, etc.), the testing phase was similar to the training phase, except for the texture floors that, from the CD stage onwards, were not neutral anymore. The number of exploratory trials at the beginning of each stage was reduced from ten to four. The maximum duration of the daily session was increased from 40 to 60 min. As in the manual version, we wanted testing to continue each day until the mouse was willing to work; to this aim, when the mouse did not express any choice while being confined within a chamber for a period of 10 min, the sliding door automatically lowered allowing the mouse to shuttle to the opposite chamber and start a new trial. However, if a similar occurrence happened a second time within the same daily session, testing was automatically terminated. The testing session also ceased if the animal failed to shuttle to the opposite chamber once the sliding door had been lowered for a period of 5 min.

As in the manual version, in order to complete each stage, mice had to reach a criterion of eight correct discriminations out of ten consecutive trials (the four exploratory trials were included in the scoring). Animals that failed to reach this criterion during the daily sessions available for each stage were excluded from the testing phase.

### Statistical analyses

For all parameters investigated, the sample size was estimated based on a sample size calculation using data from previous experiments, employing the free software G*Power 3.1, considering α = 0.05, 1 − β = 0.80 and a large effect size (Cohen’s f = 0.75).

In both the manual and the automated version, we scored the total number of trials necessary to complete each stage of the task, which provides a measure of cognitive flexibility. Errors to criterion were also counted to have an additional measure of individual performance.

Firstly, we conducted separated analyses of the manual and the automated versions to investigate between-strain differences within each stage by performing stage by stage analyses of variance (ANOVA) with strain (2 levels: CD1 vs. C57BL/6 in Experiment 1) or group (2 levels: VEH vs. CORT in Experiment 2) as between-subjects factor.

Secondly, we conducted combined analysis of the manual and the automated versions. As measures (i.e., number of trials, number of errors) obtained in the two versions of the task were on different scales (see the “Results” section for additional details), and therefore could not be directly compared, we devised two distinct approaches: (iii) the z-score normalization of both trials and errors based on the version, intended to minimise the effect of this variable; (iv) the calculation of a variable called efficiency, which allowed to evaluate whether the two versions had a differential impact on the proportion of errors committed.

For the z-score normalization we used the following formula: z_i_ = (x_i_ − $\overline{x}$)/s, where z_i_ is the resulting z score, x_i_ the value to be standardised, $\overline{x }$ the sample mean (considering the two versions separately) and s the sample standard deviation (considering the two versions separately). The efficiency to complete each stage was calculated using the following formula: [Trials performed to reach the criterion/(Trials performed to reach the criterion + Errors committed)] × 100.

The number of trials and errors following standardisation and the efficiency were analysed by performing a repeated measures ANOVA with strain (2 levels: CD1 vs. C57BL/6 in Experiment 1) or group (2 levels: VEH vs. CORT in Experiment 2) and version (2 levels: manual, automated) as between-subjects factors and stage (4 levels: CD, CDR, IDS, EDS) as within-subject factor. For multiple post-hoc comparisons we opted for the Tukey test as it is a conservative post-hoc approach (i.e. the calculation of the minimal significant difference is protected against false positive findings).

Finally, Fisher's exact test was performed to evaluate potential within-group differences in the number of animals that failed to complete the manual vs. automated ASST.

All statistical analyses were conducted using the software StatView 5.0 (Abacus Concepts, USA). Data are always expressed as mean ± SEM. Significance level was set at P ≤ 0.05.

### Supplementary Information

Supplementary Information.

## Data Availability

Raw data are available, to interested scientists, upon request to the corresponding author.
