# Supplementary material for: Automation at the service of the study of executive functions in preclinical models
Source: Sci Rep. 2023 Oct 6;13:16890. doi: 10.1038/s41598-023-43631-8 (PMC10558442; doi:10.1038/s41598-023-43631-8)
Supplement: Supplementary file 1 — Supplementary Information. [file 41598_2023_43631_MOESM1_ESM.docx]

**SUPPLEMENTARY MATERIAL**

**2. RESULTS**

**2.1. Separated analysis of manual and automated versions**

***2.1.1. Experiment 1***

CD1 and C57BL/6 mice readily learned to dig for food rewards and performed similarly in the first stage of the task (manual: F(1,16)=0.55, P=0.469 and F(1,16)=2.97, P=0.104 for trials and errors respectively; automated: F(1,10)=0.72, P=0.415 and F(1,10)=1.18, P=0.303 for trials and errors respectively), which required only a simple discrimination on a stimulus dimension.

CD1 mice learned the manual SD stage in 13.11 ± 0.98 trials (making 3.22 ± 0.55 errors) and C57BL/6 mice in 14.78 ± 2.03 trials (making 5.44 ± 1.17 errors). As for the automated version, CD1 mice learned the SD stage in 48.50 ± 17.98 trials (making 23.17 ± 10.28 errors) and C57BL/6 mice in 64.33 ± 4.77 trials (making 34.67 ± 2.58 errors).

***2.1.2. Experiment 2***

CORT treatment hampered the performance of stressed mice in the first stage of the task in the manual version only (manual: F(1,22)=7.85, P=0.010 and F(1,2)=6.99, P=0.015 for trials and errors respectively; automated: F(1,15)=3.14, P=0.097 and F(1,15)=2.99, P=0.104 for trials and errors respectively).

VEH mice learned the manual SD stage in 11.67 ± 0.65 trials (making 2.75 ± 0.48 errors) and CORT mice in 14.67 ± 0.85 trials (making 4.25 ± 0.30 errors). As for the automated version, VEH mice learned the SD stage in 108.70 ± 23.09 trials (making 52.90 ± 11.90 errors) and CORT mice in 51.86 ± 19.23 trials (making 24.43 ± 9.73 errors).

**Supplementary table 1.** F- and P-Value of the separated analysis for manual and automated versions.

| **Experiment 1** | | | | | | | | | | | |  |
| --- | --- | --- | --- | --- | --- | --- | --- | --- | --- | --- | --- | --- |
|  |  | **Trials** | | | | | **Errors** | | | | | |
| **Effect** | **Version** | SD | CD | CDR | IDS | EDS | SD | CD | CDR | IDS | EDS | |
| Strain | Manual | F(1,16)=0.55P=0.469 | F(1,16)=0.82P=0.379 | F(1,16)=4.79P=0.044 | F(1,16)=2.72P=0.118 | F(1,16)=0.01P=0.913 | F(1,16)=2.97P=0.104 | F(1,16)=0.82P=0.379 | F(1,16)=6.10P=0.025 | F(1,16)=6.94P=0.018 | F(1,16)=0.07P=0.797 | |
|  | Automated | F(1,10)=0.72P=0.415 | F(1,10)=1.20P=0.298 | F(1,10)=6.49P=0.029 | F(1,10)=3.29P=0.100 | F(1,10)=1.34P=0.274 | F(1,10)=1.18P=0.303 | F(1,10)=1.25P=0.289 | F(1,10)=5.69P=0.038 | F(1,10)=2.92P=0.118 | F(1,10)=1.20P=0.299 | |
| **Experiment 2** | | | | | | | | | | | |  |
|  |  | **Trials** | | | | | **Errors** | | | | | |
| **Effect** | **Version** | SD | CD | CDR | IDS | EDS | SD | CD | CDR | IDS | EDS | |
| Treatment | Manual | F(1,22)=7.85P=0.010 | F(1,22)=0.92P=0.348 | F(1,22)=0.89P=0.357 | F(1,22)=6.03P=0.022 | F(1,22)=3.94P=0.06 | F(1,22)=6.99 P=0.015 | F(1,22)=0.85P=0.367 | F(1,22)=1.25P=0.276 | F(1,22)=3.13P=0.091 | F(1,22)=0.85P=0.366 | |
|  | Automated | F(1,15)=3.14P=0.097 | F(1,15)=1.95P=0.183 | F(1,15)=2.59P=0.129 | F(1,15)=6.22P=0.025 | F(1,15)=0.01P=0.921 | F(1,15)=2.99P=0.104 | F(1,15)=1.71P=0.211 | F(1,15)=2.44P=0.139 | F(1,15)=5.46P=0.034 | F(1,15)=0.04P=0.84 | |

**Supplementary table 2.** F- and P-Value of the combined analysis for manual and automated versions following standardisation.

| **Experiment 1** | | |
| --- | --- | --- |
| **Effect** | **Trials** | **Errors** |
| Stage | F(3,78)=0.18, P=0.910 | F(3,78)=0.13, P=0.941 |
| Version | F(1,26)=0.05, P=0.832 | F(1,26)=0.06, P=0.813 |
| Strain | F(1,26)=0.01, P=0.940 | F(1,26)=0.002, P=0.962 |
| Stage × version | F(3,78)=0.22, P=0.880 | F(3,78)=0.14; P=0.932 |
| Stage × strain | F(3,78)=7.08; P<0.001 | F(3,78)=8.06, P<0.001 |
| Strain × version | F(1,26)=0.09, P=0.768 | F(1,26)=0.13, P=0.723 |
| Stage × version × stage | F(3,78)=0.30, P=0.823 | F(3,78)=0.20, P=0.896 |
| **Experiment 2** | | |
| **Effect** | **Trials** | **Errors** |
| Stage | F(3,111)=0.05, P=0.99 | F(3,111)=0.04, P=0.99 |
| Version | F(1,37)=0.03, P=0.863 | F(1,37)=0.02, P=0.88 |
| Treatment | F(1,27)=6.29, P=0.017 | F(1,27)=6.02, P=0.018 |
| Stage × version | F(3,111)=0.05, P=0.986 | F(3,111)=0.04; P=0.988 |
| Stage × treatment | F(3,111)=3.97, P=0.001 | F(3,111)=1.92, P=0.131 |
| Treatment × version | F(1,37)=0.29, P=0.592 | F(1,37)=0.55, P=0.462 |
| Stage x treatment × version | F(3,111)=0.68, P=0.564 | F(3,111)=1.02, P=0.388 |

**Supplementary table 3.** F- and P-Value of the combined analysis for efficiency.

| **Experiment 1** | |
| --- | --- |
| Stage | F(3,78)=6.87, P<0.001 |
| Version | F(1,26)=191.74, P<0.001 |
| Strain | F(1,26)=1.22, P=0.280 |
| Stage × version | F(3,78)=2.11, P=0.106 |
| Stage × strain | F(3,78)=3.72; P=0.015 |
| Strain × version | F(1,26)=0.53, P=0.475 |
| Stage × version × stage | F(3,78)=1.80, P=0.154 |
| **Experiment 2** | |
| Stage | F(3,111)=1.37, P=0.255 |
| Version | F(1,37)=167.365, P<0.001 |
| Treatment | F(1,37)=2.86, P=0.099 |
| Stage × version | F(3,111)=1.37, P=0.276 |
| Stage × treatment | F(3,111)=0.17, P=0.914 |
| Treatment × version | F(1,37)=1.7, P=0.201 |
| Stage × treatment × version | F(3,111)=0.13, P=0.942 |

**4. METHODS**

**4.2. Animals and experimental design**

***4.2.2. Experiment 2***

To dissolve CORT in water we used 2-hydroxypropyl-β-cyclodextrin (Sigma-Aldrich, Missouri, USA) at a dose of 4.5 mg/ml. The solution was exposed to a pre-treatment with sonication for one hour, to promote the dissolution of the corticosterone crystals, and then stirred with a magnetic stirrer for two hours. The VEH group received a solution of 2-hydroxypropyl-β-cyclodextrin at a dose of 4.5 mg/ml dissolved in drinking water. Both the VEH and the CORT solutions were changed approximately every 7 days; the CORT solution was administered in opaque-bottles to prevent the light-induced degradation of CORT.

To confirm that CORT administration was able to increase plasma CORT concentrations, blood samples were collected between 14:00 and 15:00. Briefly, mice were taken from their home-cage, carried by a familiar experimenter to an adjacent room and bled from the tail (approximately 0.03 ml collected into prechilled ethylenediamine tetraacetic acid (EDTA)-coated tubes; Microvette^®^, Sarstedt, Sevelen, Switzerland) by tail incision (Fluttert et al., 2000). The time elapsed between the experimenter entering the room and the completion of baseline blood sampling was less than 3 min. Samples were cool centrifuged (2500 rpm for 20 min) and the plasma was stored at −80 °C until assayed. Corticosterone was measured using a commercially available Corticosterone Enzyme-Linked Immunosorbent Assay (ELISA) kit (Enzo Life Sciences, Inc.).

Fluttert, M., Dalm, S., Oitzl, M. S. A refined method for sequential blood sampling by tail incision in rats. *Lab Anim* **34**, 372–378 (2000).
